# ACCELERATED PACE OF BIOLOGICAL AGING IN PARTICIPANTS WITH LOW LEVELS OF SELENIUM BIOMARKERS IN BERLIN AGING STUDY II

**DOI:** 10.1093/geroni/igae098.2617

**Published:** 2024-12-31

**Authors:** Valentin Vetter, Kamil Demircan, Jan Homann, Michael Mülleder, Markus Ralser, Lars Bertram, Lutz Schomburg, Ilja Demuth

**Affiliations:** Charité - Universitätsmedizin Berlin, Berlin, Berlin, Germany; Charité - Universitätsmedizin Berlin, Berlin, Berlin, Germany; University of Münster, Münster, Nordrhein-Westfalen, Germany; Charité - Universitätsmedizin Berlin, Berlin, Berlin, Germany; Charité - Universitätsmedizin Berlin, Berlin, Berlin, Germany; University of Lübeck, Lübeck, Schleswig-Holstein, Germany; Charité - Universitätsmedizin Berlin, Berlin, Berlin, Germany; Charité - Universitätsmedizin Berlin, Berlin, Berlin, Germany

## Abstract

Low levels of selenium in blood were shown to be associated with adverse health outcomes and are discussed as anti-aging interventions by some investigators. Inter-individual differences in the aging process can be quantified by epigenetic clocks (DNA methylation age, DNAmA). In this study, we analyzed 1,568 participants of the Berlin Aging Study II (BASE-II, mean age +/- SD: 68.8 +/- 3.7 years, 51% women). DNAmA, and its deviation from chronological age, DNAmA acceleration (DNAmAA), were measured from DNA methylation data derived from Illumina’s “MethylationEPIC” array via the Horvath, GrimAge, and DunedinPACE clock algorithms. Selenium was measured in serum samples via total reflection X-ray fluorescence (TXRF) spectroscopy. Selenoprotein P (SELENOP) was measured with ELISA. GPX3 was assessed as part of a larger set of proteins by mass spectrometry. A statistically significantly higher pace of ageing (using the DunedinPACE clock) was observed for participants with deficient selenium levels (< 90 μg/L). This association persisted after adjustment for age, sex, BMI, smoking, and genetic ancestry in linear regression (β=-0.02, SE=0.01, p= 0.012; n=757). Similarly, participants in the highest quartile of SELENOP levels showed a statistically significantly lower pace of ageing compared to the lowest quartile (DunedinPACE, β=-0.03, SE=0.01, p=0.007; n=740). Participants within the lowest quartile of GPX3 levels showed statistically significantly higher rates of biological ageing compared to the participants in the third quartile (HorvathDNAmAA, p=0.04), the fourth quartile (GrimAgeDNAmAA, p=0.003) or both quartiles combined (DunedinPACE, p=0.002). In conclusion, our findings support previous reports of potentially detrimental effects of low levels of selenium biomarkers.

